# Long-term uptake rate of a breast cancer screening program in Fukushima, Japan, following the 2011 Triple Disaster: a retrospective observational study

**DOI:** 10.1038/s41598-023-33717-8

**Published:** 2023-04-24

**Authors:** Akihiko Ozaki, Hiroaki Saito, Yudai Kaneda, Toyoaki Sawano, Yoshitaka Nishikawa, Michio Murakami, Masaharu Tsubokura, Kei Hirai, Hiromichi Ohira

**Affiliations:** 1 Department of Breast and Thyroid Surgery, Jyoban Hospital of Tokiwa Foundation, Iwaki, Fukushima Japan; 2 Research Center for Community Health, Minamisoma Municipal General Hospital, Minamisoma, Fukushima Japan; 3 Department of Gastrointestinal Tract Surgery, Fukushima Medical University, Fukushima, Japan; 4 Department of Internal Medicine, Soma Central Hospital, Soma, Fukushima Japan; 5 Department of Radiation Health Management, Fukushima Medical University School of Medicine, Fukushima, Fukushima Japan; 6 Hokkaido University School of Medicine, Sapporo, Hokkaido Japan; 7 Department of Surgery, Jyoban Hospital of Tokiwa Foundation, Iwaki, Fukushima Japan; 8 Department of Internal Medicine, Hirata Central Hospital, Hirata Village, Fukushima, Japan; 9 Department of Health Risk Communication, Fukushima Medical University School of Medicine, Fukushima, Fukushima Japan; 10 Department of Clinical Psychology, Graduate School of Human Sciences, Osaka University, Osaka, Japan; 11 Department of Surgery, Minamisoma Municipal General Hospital, Minamisoma, Fukushima Japan; 12 Department of Breast Surgery, Jyoban Hospital of Tokiwa Foundation, 57 Kaminodai, Jyoban-Kamiyunaga-Yamachi, Iwaki, Fukushima Japan; 13 Present Address: Center for Infectious Disease Education and Research, Osaka University, Suita, Osaka Japan

**Keywords:** Cancer, Natural hazards

## Abstract

Little is known about how crises might affect the long-term uptake of breast cancer screening programs. This study aimed to clarify the long-term trend of breast cancer screening program uptake in Minamisoma City following the 2011 Triple Disaster in Fukushima, Japan (earthquake, tsunami, and nuclear disaster), and to evaluate the factors associated with this uptake. This study retrospectively analyzed data from the Basic Resident Registry and Breast Cancer Screening Program in Minamisoma City following the Triple Disaster. We calculated the annual breast cancer screening uptake rate for women aged 40–74 years who were of an even-numbered age at the end of each fiscal year and the incidence of at least one instance of uptake of the breast cancer screening initiative during the biennial intervals. We further performed cross-sectional and longitudinal regression analyses for the biannual screening uptake and investigated its associated factors. Breast cancer screening participation rates were 19.8% and 18.2% in 2009 and 2010, respectively. They decreased to 4.2% in 2011, and gradually increased thereafter, reaching the pre-disaster level of 20.0% in 2016. Similar but longer decrease of the uptake was observed in the biannual screening uptake rate. No pre-disaster screening uptake between 2009 and 2010, those living alone, or those who were evacuated, were factors that were found to be associated with non-uptake of the breast cancer screening program following the 2011 disaster. This study showed a long-term decline in breast cancer screening uptake in the area affected by the Triple Disaster, which was the most severe among those under evacuation, those who were isolated, and those without previous uptake. The insights emerging from this study could be used to increase awareness of this issue and establish potential countermeasures.

## Introduction

Breast cancer is one of the most common cancers in women, with an estimated 2.3 million newly cases in 2020 worldwide^1^. Early diagnosis and treatment of breast cancer are important, and the benefits of regular screening programs, namely mammography, have been established mainly in high-income countries worldwide^2,3^ reducing the risk of death from breast cancer by an estimated 15%^4^. However, breast cancer screening uptake varies between high-income counties^5^, and even within countries, there are significant disparities in uptake levels^6^. Various individual and external factors are related to non-uptake of breast cancer screening programs^6–9^.

There has been a growing concern over the past few years about the effects of disasters and crises on breast cancer screening, an argument that has been further enhanced by the novel coronavirus disease 2019 (COVID-19) pandemic^10,11^. Before this pandemic, only a few studies had evaluated the effect of disasters and crises on cancer screening programs as a whole^12,13^. However, since the beginning of the global coronavirus disease (COVID-19) pandemic in November 2019, a global decline in screening rates for breast cancer and other cancers has been reported^14–18^. Thus, the significance of considering the impact of extrinsic factors on breast cancer screening participation is escalating, owing to the burgeoning incidence of calamities and emergencies.

Within this framework, a comprehensive evaluation of the enduring consequences of the Great East Japan Earthquake, together with the subsequent tsunami and Fukushima Daiichi Nuclear Power Plant (FDNPP) catastrophe, commonly known as the “3.11 Triple Disaster”, on breast cancer screening engagement trends in the neighboring regions may constitute a significant and informative subject of analysis. Following the 3.11 Triple Disaster, physical damage to the local medical infrastructure was limited, but the number of medical staff decreased due to the large-scale evacuation^19,20^. Furthermore, some local residents refrained from undergoing medical consultations due to either fear of radiation exposure or decreased prioritization of their own long-term health^10^.

Minamisoma City in particular experienced this phenomenon, as a municipality located at the northern edge of the evacuation zone that was set up around the nuclear power plant following the disaster^21^. The central part of Minamisoma City (Haramachi Ward) was outside the evacuation zone, and the administrative function of the city remained. However, the evacuation order was issued to the southern third of the city (Odaka Ward), and this evacuation order lasted until July 12, 2016. Consequently, multiple medical institutions were closed following the disaster. The city experienced health concerns related to breast cancer, as well as other cancers. For example, previous studies have suggested delays in initial medical consultations for patients with symptomatic breast cancer, and an increased proportion of patients with advanced breast cancer in the region due to these delays^10,22^. However, information is limited regarding the uptake of breast cancer screening programs among the local residents following the disaster.

This study aimed to clarify the changes that took place in the breast cancer screening uptake rate in Minamisoma City following the 3.11 Triple Disaster, and identify the factors associated with these changes. The results of this study will not only elucidate the long-term health hazards associated with cancer among the residents of Minamisoma City and the broader Fukushima Prefecture, but will also potentially provide important insights into future large-scale crises.

## Materials and methods

### Locations and study setting

Minamisoma City, Fukushima Prefecture, is located 10–40 km north of the FDNPP and consists of three districts—Odaka, Haramachi, and Kashima Ward—listed here in order of proximity to the plant. The earthquake and resulting tsunami that occurred on March 11, 2011 triggered fear that a hydrogen explosion would occur inside the FDNPP in the evening. On March 12, a hydrogen explosion did occur in the plant’s reactor. As a result, the government issued an evacuation order within a 20-km radius of the plant, which extended to Odaka Ward, and on March 15, a shelter-in-place order was issued for the 20–30 km area. On April 22, the evacuation order was revised to forbid entry into areas, where radiation levels of 20 mSv or more per year were expected. This included Odaka Ward and part of Haramachi Ward.

In April 2012, the following areas were defined in connection to the FDNPP: the difficult-to-return zone (no entry; comprising areas where radiation levels of 50 mSv or more per year were expected); the restricted residence area (temporary return allowed but no accommodation; comprising areas where radiation levels of 20–50 mSv per year were expected); and the zone in preparation for lifting of the evacuation order (temporary return allowed, but no accommodation). Restrictions on areas other than the difficult-to-return zone were removed in July 2016.

As a general rule, the breast cancer screening program in Minamisoma City is open to women aged 40 years or older at the end of the fiscal year (March 31 of each year), and there is no upper limit for age. The fee for each test is 1000–1500 JPY (approximately 10 USD), and the tests are conducted at medical institutions and healthcare centers in the city. The city office also sends free coupons to those aged 40, 45, 50, 55, and 60 at the end of the fiscal year, in order to increase the uptake of this program.

The details of the Minamisoma City screening program are presented below. Invitation letters were sent to eligible women in January, before the start of the fiscal year (April), and their intentions were confirmed by February. With an invitation letter, women could select whether to go to a medical institution within Minamisoma City or to an outreach screening program elsewhere, conducted by a breast cancer screening company. Based on their requests, the entities in charge of the screening program sent out detailed schedules between August and September, and the screenings were conducted between September and March. In principle, the breast cancer screening program is available to those who respond to the invitation letter and express their wish to participate before the beginning of the fiscal year. However, it is also available for those who originally did not intend to participate, as long as they are of an even-numbered age at the end of the year and/or had received free coupons for that year.

In 2008, the Ministry of Health, Labour, and Welfare issued a notice that, as a general rule, breast cancer screening should only be conducted only using mammography^23^, but that local governments that are responsible for breast cancer screening programs are allowed to use their own discretion regarding conducting the most appropriate test for each situation. Minamisoma City has therefore adopted a combined program of physical examination and mammography^24^.

Breast cancer screening persisted in Minamisoma City, even in the aftermath of the 2011 Triple Disaster. During the 2011 fiscal year, eligible women's intentions were verified by February. Since the 2011 fiscal year, Minamisoma City has facilitated screenings for eligible women at their evacuation site municipalities. Nonetheless, it is imperative to acknowledge that the breast cancer screening capabilities in Minamisoma City may have decreased post-disaster, as all four medical institutions that previously offered the program discontinued their services in 2011, despite not incurring significant physical damage that would have impaired their capacity to maintain the service. As a result, the solitary outreach initiative was executed during that same year. These circumstances may have significantly impacted the capacity of the breast cancer screening program, particularly during the initial year, although this impact has not yet been quantified.

In Japan, some medical institutions independently hold breast cancer screening programs in which people voluntarily undergo examinations, regardless of the breast cancer screenings conducted by their municipalities. However, these examinations were beyond the scope of this study.

### Study design and data collection

This study performed a retrospective analysis of the trends in breast cancer screening participation rates in Minamisoma City and the factors associated with uptake of the program following the 2011 Triple Disaster. We used data from the Basic Resident Registry and the Breast Cancer Screening for Minamisoma City. Data on whether a woman underwent breast cancer screening were assessed using Basic Resident Registry data for each year (year-end or new year) from 2009 to 2018. The basic resident register includes ID, sex, residential district (Odaka, Kashima, or Haramachi ward), age, number of people per household, and evacuation status since 2011. Evacuation status was categorized as evacuation outside the city, within the city, or to the resident’s home. The number of people in each household was counted as one (single), and two or more people were counted as having a co-resident.

### Outcome

The key outcome measures of this investigation were to establish the yearly rate of acceptance of breast cancer screening among females aged 40–74 years who held even-numbered ages as of the conclusion of each fiscal year, and to record the incidence of at least one instance of uptake of the breast cancer screening initiative during the biennial intervals spanning 2009–2010 and 2017–2018. Among them, we conducted regression analyses to determine the factors linked to the program’s uptake, using a biennial-based analysis, as will be further detailed in the “Data analysis” section. Our measure's criterion stipulated that female of odd-numbered ages, as of the close of each fiscal year, were eligible to partake in the program, whereby the municipal office distributes gratis vouchers to those who possess odd-numbered ages (such as 45 or 55) at the conclusion of the fiscal year. Generally, women of even-numbered ages at the end of each fiscal year were eligible to participate and received invitations, but they could decline if they had participated in the program during the previous year. Therefore, we thought that this outcome measure would offer a more precise portrayal of the broader breast cancer screening program in Japan.

We included all women between the ages of 40 and 74 years who had been residents of Minamisoma City for a minimum of 1 year during the respective 2-year periods. Although there is no age limit for breast cancer screening in Japan, this is not the case in the United States and Europe, where regional breast cancer screening initiatives originated. For instance, the US Preventive Task Force recommends against mammography screenings for individuals aged 75 years or older. Consequently, we limited our analysis to individuals below this age. Moreover, for women aged 40–49 years, the Japan Association of Breast Cancer Screening suggests performing breast cancer screening due to the relatively high incidence rate of breast cancer in this demographic in Japan. Therefore, this is the age group we chose to include in our analysis.

### Data analysis

We conducted a descriptive analysis of the annual uptake rates of breast cancer screening from 2009 to 2018, targeting the eligible population of women who were of even-numbered ages at the end of each fiscal year. Similarly, we illustrated the biannual uptake rate for the program from 2009 to 2018. As described above, the analysis was conducted using, as an outcome, whether each woman had undergone breast cancer screening at least once during the relevant 2-year period, among all the women between the ages of 40 and 74 years, who had been residents of Minamisoma City for a minimum of one year during the respective two-year periods. Next, we performed a multivariate logistic regression analysis to determine the factors associated with uptake of biennial breast cancer screening, using the same outcome measure. This analysis comprised two components: (1) a cross-sectional analysis for each biennial period and (2) a longitudinal analysis extending from the 2009–2010 period to the 2016–2018 period. In the cross-sectional analysis, we performed a logistic regression analysis using covariates (age, number of households, and evacuation status) to examine their impacts on participation rates in breast cancer screening. Covariates were evaluated in the first year of each biennial period. For the longitudinal analysis, we focused on women who resided in Minamisoma continuously from 2009 to 2018, and were eligible for breast cancer screening throughout, those aged between 40 and 65 years in 2009, and those who held registered resident certificates during these periods. Using participation in breast cancer screening during the 2009–2010 period, as well as age, household status, and evacuation status in 2011 as covariates, we conducted a logistic regression analysis to clarify the association with breast cancer screening participation after 2011. Statistical significance was set at P < 0.05. We used STATA version 15.0 (Stata Corp, Texas, USA) for all statistical analyses.

### Ethics statement

This study was approved by the ethics committees of the Minamisoma Municipal General Hospital, as well as Fukushima Medical University (approval nos. 2-20 and 3065, respectively). As this was a retrospective study, an opt-out approach was used to obtain informed consent. All analyses were performed in accordance with the Declaration of Helsinki.

### Research involving human participants and/or animals

This study was approved by the ethics committees of the Minamisoma Municipal General Hospital and Fukushima Medical University (approval nos. 2-20 and 3065, respectively).

### Informed consent

The institutional review boards of Minamisoma Municipal General Hospital and Fukushima Medical University did not require informed consent from the participants given the retrospective nature of this study.

## Results

Table 1 shows the characteristics of the target population for each year between 2009 and 2018. Characteristics such as the number of residents, ages, residence statuses, and whether they were living alone, were relatively stable during the period. Regarding evacuation status, the number of people who evacuated outside the city peaked in 2011, at 43.0% (3555/8272), and consistently decreased thereafter. By contrast, those who were in states of non-evacuation, as well as those who evacuated inside the city, increased almost consistently over the post-disaster study period (2011–2018).

**Table 1:** Characteristics of the target population for breast cancer screening in Minamisoma City in each year from 2009 to 2018.

Year	2009	2010	2011	2012	2013	2014	2015	2016	2017	2018
Number of target residents	8573	8396	8272	8068	8105	7981	8066	7790	7867	7514
Age, median (IQR)	58 (50–66)	58 (50–66)	58 (50–64)	58 (50–66)	60 (50–66)	60 (50–66)	60 (50–66)	60 (50–66)	60 (50–68)	60 (50–68)
Residence, n (%)										
Haramachi ward	5598 (65.3)	5578 (66.4)	5478 (66.2)	5380 (66.7)	5408 (66.7)	5312 (66.6)	5424 (67.2)	5268 (67.6)	5402 (68.7)	5197 (69.2)
Kashima ward	1436 (16.8)	1292 (15.4)	1364 (16.5)	1246 (15.4)	1324 (16.3)	1253 (15.7)	1351 (16.7)	1234 (15.8)	1342 (17.1)	1224 (16.3)
Odaka ward	1539 (18.0)	1526 (18.2)	1430 (17.3)	1442 (17.9)	1373 (16.9)	1416 (17.7)	1291 (16.0)	1288 (16.5)	1123 (14.3)	1093 (14.5)
Household, n (%)										
Alone	624 (7.3)	654 (7.8)	670 (8.3)	659 (8.1)	694 (8.7)	686 (8.5)	709 (9.1)	727 (9.2)	734 (9.8)	734 (9.8)
Evacuation status, n (%)										
Non-evacuation	Not applicable	Not applicable	3649 (44.1)	4706 (58.3)	4957 (61.2)	4944 (61.9)	5164 (64.0)	4962 (63.7)	5255 (66.8)	4917 (65.4)
Evacuation (inside of the city)	Not applicable	Not applicable	520 (6.3)	1245 (15.4)	1395 (17.2)	1470 (18.4)	1527 (18.9)	1568 (20.1)	1672 (21.3)	1778 (23.7)
Evacuation (outside of the city)	Not applicable	Not applicable	3555 (43.0)	2067 (25.6)	1717 (21.2)	1541 (19.3)	1360 (16.9)	1246 (16.0)	928 (11.8)	810 (10.8)
Others	Not applicable	Not applicable	548 (6.6)	50 (0.6)	36 (0.4)	26 (0.3)	15 (0.2)	14 (0.2)	12 (0.2)	9 (0.1)
Uptake of the screening, n (%)										
Participants	1700 (19.8)	1525 (18.2)	344 (4.2)	659 (8.2)	1276 (15.7)	1160 (14.5)	1410 (17.5)	1560 (20.0)	1504 (19.1)	1533 (20.4)

During the pre-disaster period, breast cancer screening uptake rates were 19.8% (1700/8573) and 18.2% (1525/8396) in 2009 and 2010, respectively. In 2011, the proportion decreased to 4.2% (344/8272), but gradually increased thereafter, to 8.2% (659/8068) in 2012, 15.7% (1276/8105) in 2013, 14.5% (1160/7981) in 2014, 17.5% (1410/8066) in 2015, and 20.0% (1560/7790) in 2016. This means that the proportion recovered to its pre-disaster level five years after the earthquake.

Table 2 shows the characteristics of the target population during the two-year interval from 2009 to 2018. Overall, no clinically significant variances were observed in the characteristics of the target population between the biennial and annual calculations. Similarly, the uptake rate of the breast cancer screening program did not exhibit any notable dissimilarities between the biennial and annual calculations, except for the biennial uptake rate, which eventually recovered to its pre-disaster level during the 2017–2018 period.

**Table 2:** Characteristics of the target population for each two-year period from 2009 to 2018.

Year	2009–2010	2011–2012	2013–2014	2015–2016	2017–2018
Number of target residents	17,110	16,546	16,269	16,049	15,137
Age, median (IQR*)	58 (49–65)	58 (50–65)	59 (49–65)	59 (49–66)	60 (50–67)
Residence, n (%)					
Haramachi ward	11,270 (65.9)	11,025 (66.6)	10,845 (66.7)	10,783 (67.2)	10,384 (68.6)
Kashima ward	2757 (16.1)	2631 (15.9)	2601 (16.0)	2611 (16.3)	2508 (16.6)
Odaka ward	3083 (18.0)	2890 (17.5)	2823 (17.4)	2655 (16.5)	2245 (14.8)
Household, n (%)					
Alone	1271 (7.4)	1303 (7.9)	1363 (8.4)	1401 (8.7)	1411 (9.3)
Evacuation status, n (%)					
Evacuation	Not applicable	9157 (55.3)	6380 (39.2)	5879 (36.6)	5115 (33.8)
Uptake of the screening, n (%)					
Participants	3649 (21.3)	1558 (9.4)	2816 (17.3)	3273 (20.4)	3235 (21.4)

*IQR: interquartile range.

The characteristics of the target population were defined over two-year periods. The variables are the statuses in the earlier years in each two-year period.

Table 3 illustrates the cross-sectional logistic regression models for breast cancer screening uptake for every 2-year period from 2009 to 2018. Throughout the study period, women who lived alone were less likely to participate in breast cancer screening than those who lived with other family members. Furthermore, those who were in evacuation statuses tended not to participate in the screening programs less often than their non-evacuated counterparts throughout the post-disaster periods.

**Table 3:** Cross-sectional logistic regression models for breast cancer screening uptake in every two-year period from 2009 to 2018.

	2009–2010		2011–2012		2013–2014		2015–2016		2017–2018	
	AOR^†^	P value	AOR^†^	P value	AOR^†^	P value	AOR^†^	P value	AOR^†^	P value
Age*	0.96 (0.92–0.99)	0.02	0.71 (0.68–0.75)	< 0.01	1.01 (0.97–1.05)	0.58	1.02 (0.98–1.06)	0.41	1.05 (1.01–1.09)	0.01
Household (reference = more than two)										
Alone	0.55 (0.47–0.65)	< 0.01	0.52 (0.40–0.67)	< 0.01	0.62 (0.52–0.73)	< 0.01	0.60 (0.51–0.70)	< 0.01	0.56 (0.48–0.65)	< 0.01
Evacuation status (reference = non-evacuation)										
Evacuation	–	–	0.90 (0.81–1.00)	0.05	0.80 (0.73–0.87)	< 0.01	0.85 (0.79–0.93)	< 0.01	0.91 (0.84–0.99)	0.03

*Odds ratios were calculated based on 10 years interval.

^†^AOR = Adjusted odds ratio; multivariable logistic regression models were developed to assess the probability of participation in the screening program for every two-year period ranging from 2009 to 2018.

Table 4 shows the outcomes of the longitudinal analyses conducted on the uptake rates of breast cancer screening for every two-year period from 2011 to 2018. A series of analyses were undertaken for 11,695 women. With regard to the cross-sectional analysis, our evaluation of household status revealed that living without any other family members was significantly linked to a reduced uptake of breast cancer screening during the post-disaster phase. Furthermore, individuals who evacuated immediately following the disaster were considerably less inclined to participate in the screening program during the 2011–2012 and 2013–2014 periods. However, no such correlations were identified for the 2015–2016 and 2017–2018 periods. The uptake of the breast cancer screening program between 2009 and 2010 was significantly linked to the general increase in the uptake of the program during the post-disaster period.

**Table 4:** Longitudinal analyses for the uptake rates of breast cancer screening in every two-year period from 2011 to 2018.

	2011–2012		2013–2014		2015–2016		2017–2018	
	AOR^†^	P value	AOR^†^	P value	AOR^†^	P value	AOR^†^	P value
Age*	0.88 (0.81–0.95)	< 0.01	1.15 (1.08–1.24)	< 0.01	1.06 (0.99–1.13)	0.09	1.07 (1.00–1.14)	0.05
Household (reference = more than two)								
Alone	0.61 (0.43–0.86)	< 0.01	0.65 (0.50–0.85)	< 0.01	0.60 (0.46–0.77)	< 0.01	0.55 (0.43–0.71)	< 0.01
Evacuation status just after the disaster (reference = non-evacuation)								
Evacuation	0.84 (0.74–0.95)	< 0.01	0.89 (0.80–0.98)	0.02	0.98 (0.89–1.08)	0.65	0.95 (0.86–1.04)	0.28
Uptake of the breast cancer screening in 2009–2010 (reference = absent)								
Yes	5.25 (4.64–5.93)	< 0.01	8.57 (7.72–9.51)	< 0.01	7.67 (6.95–8.47)	< 0.01	6.54 (5.94–7.21)	< 0.01

*Odds ratios were calculated based on 10 years interval.

^†^AOR = Adjusted odds ratio; multivariable logistic regression models were formulated to determine the likelihood of participation in the screening program for each biennial period spanning from 2011 to 2018. The data pertaining to evacuation statuses were procured from 2011, whereas the other explanatory variables used in the models were sourced from 2009 to 2010.

## Discussion

This study was conducted to clarify the changes in breast cancer screening uptake rate that took place in Minamisoma City after the 3.11 Triple Disaster, and identify the factors associated with them. As a result of annual and biannual analyses of the breast cancer screening uptake rate, we showed that it took at least five years for the breast cancer screening uptake rate to recover to its pre-disaster level, following a sharp decline in 2011 when the earthquake occurred. Living alone and being under evacuation were associated with non-uptake in breast cancer screening while a pre-disaster uptake of the program was associated with its uptake.

The most important finding of this study is that it took at least five years for breast cancer screening rates to recover to pre-disaster levels. However, this finding must be interpreted with some caution. This is because during the study period, breast cancer screening uptake rates increased in general, all over Japan. Although there are no survey results specific to municipal-sponsored breast cancer screening, the overall breast cancer screening rate for people aged 40–69 years has increased from 39.1% in 2010 to 47.4% in 2019^25^. In this respect, we may conclude that the effects of the disaster are still lingering, as the results of this study show that, as of 2018, the rate had only reached its pre-disaster level.

We previously reported comparable outcomes for local colorectal cancer screening using fecal occult blood in Minamisoma City^13^. However, there are two important differences between breast and colorectal cancer screening. Firstly, breast cancer screening requires medical practitioners to perform breast examinations and radiation technicians to perform mammography, whereas fecal occult blood testing can be conducted and submitted for analysis by the public. This implies that breast cancer screening is a more demanding program to implement than colorectal cancer screening as it requires greater medical resources. Conversely, breast cancer screening is simpler to maintain in terms of frequency, as it is a biannual program, whereas colorectal cancer screening is an annual program. Our results demonstrate a more robust impact of the 2011 Triple Disaster on local cancer screening programs, causing a long-term decline in participation rates for these two distinct types of cancer screenings.

With regard to the protracted reduction in breast cancer screening uptake rates following the disaster, it is reasonable to postulate that the cause may not be limited to the constraints of medical infrastructure, given the minimal damage to Minamisoma City. Referring to the outcomes of our regression analyses, and the regional situation following the disaster, a decrease in access to medical care due to displacement and an increase in the number of individuals living alone can be seen. Regarding the former, it was noticeable that active evacuation was associated with a more prolonged decrease in screening uptake than evacuation only immediately after the disaster. Although various options were available for this cohort, it may have been arduous for the evacuees to undergo breast cancer screening in Minamisoma City, due to psychological, economic, and physical challenges. Given the vast expanse of the Fukushima Prefecture, the scarcity of well-established infrastructure, and the establishment of evacuation zones, as well as transportation constraints following the disaster, access to breast cancer screening may have declined markedly among the local population.

The results for those living alone are consistent with those of past studies, showing the importance of family support in general and post-disaster health checkups^26,27^. Moreover, in Minamisoma City, the number of people living together in one household decreased, from an average of 3.00 in 2010 to 1.99 in 2020, due to the evacuation of many of the younger and elderly generations following the earthquake^28^, and such changes are hypothesized to have contributed to the decline in the breast cancer screening participation rate. In the future event of a major disaster, it will be necessary to provide more support to evacuees through outreach and support activities that take those who live alone into consideration, such as through individual visits by health center staff.

Longitudinal analyses indicated that an increase in breast cancer screening uptake in 2009 or 2010 resulted in a notably higher program uptake after the disaster. This correlation was similarly observed in a colorectal cancer screening analysis following the disaster^13^. Therefore, it may be inferred that those who do not participate in cancer screening programs before a disaster are also unlikely to do so after the occurrence of one. Implementing interventions to encourage participation may be a crucial method for augmenting overall program uptake.

### Implications of the study findings

We posit that these findings have multiple potential uses. Firstly, they are instrumental to understanding the impact of the 2011 Triple Disaster on local cancer screening. This study is the third of its kind, examining a unique form of cancer for which the long-term participation rate has been reported after the aforementioned disaster. All three studies found a lasting decrease in participation rates^12,13^. These outcomes enable us to investigate the long-term consequences of this drop in local cancer screenings in the future. Despite the potential increase in advanced disease, an enhanced outcome has been observed in breast cancer thus far^29^, ostensibly due to improved treatment measures that may have outweighed the negative effects of reduced participation in local breast cancer screening programs.

Second, these findings may have implications for understanding the effects of future and ongoing disasters and crises on cancer screening, although these must be approached with caution. For instance, while this study's results may be relatively simple to integrate in the event of a similar disaster occurring in Japan, it may prove more difficult to apply these findings to other disasters in different countries or regions. This may be due to potential variations in specific methods used for breast cancer screening elsewhere, as well as distinct consequences of disasters and differences in social/cultural contexts. Nevertheless, the most critical aspect of this study, as well as similar previous studies following the 2011 Triple Disaster, is the long-term decline in participation rates in the breast cancer screening program studied, rather than solely the participation rate itself. These findings may therefore prove useful in future disaster events.

It is therefore important to consider whether these findings are applicable to the current COVID-19 pandemic. However, it is premature to draw definitive conclusions from the available evidence, as the 2011 Triple Disaster and the ongoing pandemic are two distinct crises. Thus, we must closely monitor how participation rates in breast cancer screening programs fluctuate over time during the pandemic, both within and outside Japan. At this time, we have only observed the prolonged impact of the pandemic on the participation rate in breast cancer screening in Japan.

### Limitations

This study had several key limitations. First, the study was conducted only in Minamisoma City, so its generalizability is therefore limited. Given the low breast cancer participation rate, the findings may not be generalizable to other areas with higher breast cancer screening participation rates. Second, the unique Japanese framework of cancer-screening programs should be acknowledged. In Japan, the entire framework of cancer screening programs consists of programs organized by each local municipality, those provided at the workplaces of residents or their family members, and individual participation in cancer programs. Particularly, the cancer-screening program organized by each local municipality occupies only a part of the entire framework, which this study failed to capture. Nonetheless, we believe that our study has important implications for the body of evidence regarding the effects of disasters on cancer as described above.

## Conclusion

This study assessed extended breast cancer screening data in Minamisoma City subsequent to the 2011 Triple Disaster. The uptake rate of the breast cancer screening program exhibited a notable decrease in the years following the disaster. It was observed that individuals who were evacuated, as well as those residing alone in the area, showed reduced propensities to participate in the Minamisoma screening program. Conversely, higher uptake of the screening program prior to the disaster was linked to an increased uptake following the disaster.

Considering that this study makes a noteworthy contribution to previous studies that reported the long-term impacts of the 2011 Triple Disaster on local colorectal and cervical cancer screening programs, we recommend the implementation of effective countermeasures to mitigate the impacts of disasters on cancer screening programs in the future. However, we lack a complete understanding of the effects and variations of disasters on cancer screening in the affected areas. Therefore, global efforts should be intensified to conduct further studies on this topic.

## Data Availability

The data supporting the findings of this study are available from the corresponding author, Akihiko Ozaki, upon reasonable request.
